# Relationship building in pediatric research recruitment: Insights from qualitative interviews with research staff

**DOI:** 10.1017/cts.2022.469

**Published:** 2022-10-03

**Authors:** Stephanie A. Kraft, Kathryn M. Porter, Tara R. Sullivan, Emily E. Anderson, Nanibaa’ A. Garrison, Laura Baker, Jodi M. Smith, Elliott M. Weiss

**Affiliations:** 1 Department of Pediatrics, University of Washington School of Medicine, Seattle, WA, USA; 2 Treuman Katz Center for Pediatric Bioethics, Seattle Children’s Research Institute, Seattle, WA, USA; 3 Stanford University, Stanford, CA, USA; 4 Neiswanger Institute for Bioethics, Loyola University Chicago Stritch School of Medicine, Maywood, IL, USA; 5 Institute for Society and Genetics, University of California, Los Angeles, Los Angeles, CA, USA; 6 Institute for Precision Health, David Geffen School of Medicine, University of California, Los Angeles, Los Angeles, CA, USA; 7 Division of General Internal Medicine and Health Services Research, Department of Medicine, David Geffen School of Medicine, University of California, Los Angeles, Los Angeles, CA, USA; 8 Research Integration Hub, Seattle Children’s Research Institute, Seattle, WA, USA

**Keywords:** Recruitment, clinical trials, interviews, clinical research, equity, informed consent, workforce, qualitative

## Abstract

**Introduction:**

Clinical research staff play a critical role in recruiting families for pediatric research, but their views are not well described. We aimed to describe how pediatric research staff build trusting research relationships with patients and their families.

**Methods:**

We interviewed research staff at one pediatric research institution and its affiliated academic medical center between November 2020 and February 2021. Staff were eligible if they conducted participant recruitment, consent, and/or enrollment for clinical research. We developed our semi-structured interview guide based on a framework for trusting researcher-community partnerships.

**Results:**

We interviewed 28 research staff, with a median age of 28 years (range 22–50) and a median of 5 years of experience (range 1–29). Interviewees identified factors relevant to relationship building across three levels: the individual staff member, the relational interaction with the family, and the institutional or other structural backdrop. Individual factors included how staff developed recruitment skills, their perceived roles, and their personal motivations. Relational factors spanned four stages of recruitment: before the approach, forming an initial connection with a family, building the connection, and following up. Structural factors were related to access and diversity, clinical interactions, and the COVID-19 pandemic.

**Conclusions:**

Research staff discussed tensions and supports with various actors, challenges with the integration of research and clinical care, the importance of voluntariness for building trust, and multiple contributors to inequities in research. These findings reveal the importance of ensuring research staff have a voice in institutional policies and are supported to advocate for patients and families.

## Background

Clinical research staff, including research coordinators, clinical research assistants, and research nurses, are key actors in the recruitment process for pediatric clinical research. Staff have a range of participant-facing responsibilities, including recruitment, consent, scheduling, and data collection [1]. Research staff frequently serve as the first introduction to clinical research for patients and their families and sometimes the family’s primary point of contact throughout the study.

Interactions between research staff and prospective participants and their families can affect the success of pediatric research. A recent survey found that parents who enrolled in a neonatal clinical trial were more likely than non-enrollees to report a positive initial impression of the research staff [2]. Positive staff-family relationships are supported by approaches such as respectful communication, attention to patient and family needs, and candor about the realities of study participation,[3] as well as awareness of potential challenges such as language barriers or misgivings about research [4]. Barriers to enrollment may arise if the approach is poorly timed [5], the family perceives a lack of empathy from the recruiter [5], the study and its potential benefits are not described clearly and concisely [6], or recruiters have biases that lead them to view underrepresented participants as non-ideal candidates for participation [7]. Additionally, structural barriers often shape these personal interactions, for example gaps in interpretation and language translation services [8].

While research staff play a critical role in building research relationships, their views on the relationship-building process are sparsely described, particularly in the pediatric context. This study describes the perspectives of pediatric research staff about how they engage with patients and families, including barriers and facilitators, and offers a framework for understanding the process of relationship building throughout research recruitment.

## Methods

### Overview

We conducted one-on-one interviews with pediatric research staff involved with study recruitment to allow for nuanced exploration of experiences and perspectives [9]. This study was approved by the Seattle Children’s Institutional Review Board.

### Recruitment

Eligible participants were research staff at a single pediatric research institution and its affiliated academic medical center who were involved with participant recruitment, consent, and/or enrollment. We emailed invitations to (a) members of a clinical research coordinator core service, (b) subscribers to an internal professional development listserv, and (c) individuals we knew to be working in research recruitment in clinical settings. Participants received a $25 incentive for their time.

### Interviews

We developed a semi-structured interview guide (Supplementary Appendix) based on a framework for researcher-community partnerships that addressed effective communication, respectful relationships, committed partnerships, methods to resolve problems, and sustainability [10]. We conducted two pilot interviews and revised the guide for clarity and length.

One member of our team (KMP) conducted all interviews via Microsoft Teams between November 2020 and February 2021. Interviews lasted approximately 60 minutes. We evaluated for theoretical saturation throughout the interview process, including assessing for distinctions across clinical settings, and ended recruitment when additional interviews no longer produced new themes. Audio recordings of interviews were professionally transcribed, de-identified, and verified for accuracy. Transcripts were uploaded to Dedoose [11] for data management and analysis.

### Analysis

We developed a codebook using inductive and deductive techniques [12,13] focused on the five domains in the interview guide, followed by an iterative process of open coding and codebook revision whereby three members of our team (SAK, KMP, EMW) independently coded transcripts then met to reach consensus. After repeating this process and reviewing with the full study team, we revised our codebook to reflect three levels of relationship-building factors, with sub-codes describing specific factors within each level (Supplementary Material). Two trained coders (KMP and TRS) then coded remaining transcripts, first together until they were consistently in agreement on 80% or more of code applications, then independently with periodic dual coding to maintain consistency. With coding completed, interview excerpts were grouped by code and each summarized. We reviewed these summaries through iterative team discussions to identify and refine preliminary themes.

We shared our preliminary themes with a subset of interviewees for verification through the process of member checking, an approach used to ensure the research team’s interpretation of the data aligns with research participants’ experiences [14]. Interviewees were offered an additional $50 to join a 60-minute webinar that included presentation of our preliminary results followed by discussion and feedback. Eleven interviewees joined one of three discussions held between August and September 2021.

## Results

### Interviewee Characteristics and Overview

We completed 28 interviews, representing 72% of the 39 individuals who either responded to our general outreach (*n* = 21) or whom we contacted directly (*n* = 18). Of these 39, 5 did not respond, 4 did not meet inclusion criteria, and 2 declined. Table 1 shows self-reported interviewee demographics.

**Table 1.** Interviewee demographics (n = 28)

Characteristic	*n* (%)
Median years of experience	5 (range 1–29)
Median age in years	28 (range 22–50)
Gender	
Female Male Non-binary	21 (75%) 6 (21%) 1 (4%)
Race and/or ethnicity	
White, not Hispanic or Latino/a/x Asian Hispanic or Latino/a/x, no racial category identified 2+ racial or ethnic categories identified	17 (61%) 6 (21%) 3 (11%) 2 (7%)
Highest degree earned	
Bachelors Masters Graduate/PhD	19 (68%) 6 (21%) 3 (11%)
Primary research setting	
Specialty clinic Social/behavioral Clinical research core Emergency department Neonatal or pediatric intensive care	10 (36%) 7 (25%) 5 (18%) 3 (11%) 3 (11%)

Interviewees described factors that influence how they form research relationships with patients and their families at the level of the individual staff member, relational interactions with families, and institutional or other structural backdrops. We did not identify major differences across clinical settings but note any minor distinctions below.

### Individual Staff Member

Individual factors affecting relationship building included how interviewees developed recruitment skills, their perceived roles, and personal motivations for their work. Table 2 shows exemplar quotes.

**Table 2.** Individual factors affecting relationship building: codes and exemplar quotes

Factor	Exemplar quote and primary research setting*
Developing recruitment skills	I was mentored by a really great PI [principal investigator], who kind of showed me the ropes on how to interact with families, but I think apart from just the very brief beginning, it was mostly you know I volunteered a lot in hospitals. …. I kind of already had an idea how practitioners and providers spoke to families, especially observing the doctors, and shadowing and all that stuff. *(Emergency department)*
Perception of role	I see my job as an educator and, ultimately, the decision is not mine to make, but I consider a good job done when I feel like a family has been given as much information as they need to make their decision. I really see it as two groups of people on equal footing. *(Clinical research core)*
Personal motivations	My first kind of position in research was more with immigrants and refugees. So a very vulnerable group, but I mean it was a very personal cause. Both of my parents are refugees. … We were trying to assess their level of comfort accessing healthcare in the United States as refugees, so it’s already a very vulnerable, volatile context, and this was also in 2017. So you know, the political climate at the time wasn't the nicest. *(Specialty clinic)*

* Interview code numbers omitted for interviewee privacy.

Most interviewees reported having developed recruitment skills by observing other recruiters, following standard operating procedures, or drawing on communication skills from prior jobs. Few reported receiving formal training. Interviewees perceived their roles as supporting participants by maintaining connection and continuity, providing transparent information, being a comforting and friendly presence, and being sensitive to families’ timing and circumstances. Some also discussed their role as advancing research, promoting diversity, and collaborating with other coordinators to advance their collective learning. Several interviewees discussed personal motivations for their work, including advancing equity in research, interacting with children and families, and training other coordinators to pay forward the support they received.

### Relational Interactions

Relational factors spanned four stages of recruitment: before the approach, meeting a family and forming an initial connection, building a connection and discussing the study, and following up. Table 3 shows exemplar quotes.

**Table 3.** Relational factors affecting relationship building: codes and exemplar quotes

Factor	Exemplar quote and primary research setting*
**Pre-approach**	
Determining when and whether to approach	I will not go in, and this will be usually the nurse or the clinical team will tell me, if a parent is getting upset, cause these can be long days for parents and they can also get some bad news, or good news or whatever, but it is really important that the care team gets the most out of their interaction with the family. *(Specialty clinic)*
**Initial connection**	
Approach to communication	I think to begin, it’s always really important for families to understand particularly if you only have a few moments to really communicate with them for that first approach. I think it’s important for them to understand who you are and what you’re there to do. You don't just want to jump right into the spiel about the study. I think they first need to understand what is it really that you may need from them. That’s kind of the first part of maintaining a respectful relationship. *(Clinical research core)*
Style or persona	I always try to greet with maybe the part of the day: ‘Hi, good morning/afternoon/evening,’ things like that, and then maybe a gentle smile, but not too forceful because I feel like you could be excessively chipper sometimes, and then depending on the clinical situation, it’s maybe uncalled for. *(Social/behavioral)*
Finding common ground	I lived in [region] twice in my life … so sometimes I throw that in there. It’s a commonality and then it’s because then we get to talk about [continent] and it’s wonderful for me, them, and we’re back on the homeland. *(Intensive care)*
Clarifying role	Just my being a part of the [hospital] family can make me important, or people think that I’m a clinician or something, so I have to be very careful and be very clear with them of what my role is and credentials are and my educational background sometimes. I have to make it very plain that I’m not a clinician. *(Specialty clinic)*
Names, pronouns, and pronunciation	One of the things we try to do with all of our research studies is asking patients and families about their preferred names and gender pronouns, just to respect different identities, different nicknames, even asking or verifying that we’re pronouncing names correctly is another part of it. *(Specialty clinic)*
Families’ perception of coordinator	I think sometimes my age or appearance can sometimes lead some folks to be skeptical of my abilities. I think that’s one barrier that I face, but it’s something that you eventually overcome, once you get to know me and work with me. *(Clinical research core)*
Personal biases	I know I could definitely say things that would offend other groups, based on just my upbringing and the way that I communicate with people, if I don't choose my words carefully, and that’s true for everybody, but I think that’s an important part of building trust. *(Specialty clinic)*
Families who use a language other than English	I can get a better sense of who they are, and there have been times where, for example, from past studies, if the family speaks Spanish … we would just end up speaking Spanish just to feel much more comfortable, especially for them. *(Specialty clinic)*
**Building connection**	
Accommodating family needs	I also think flexibility can be a way of showing respect. Our patients and families have different needs at different times. … I’m happy to meet with a family a weekend, or in the evening, if that works better for them, or I can schedule a pick up for study materials for the [study] test kit, if that makes them easier, and I want to respect in that way that like I’m a part of their whole experience, but I am not the most important part. The most important part is that they are navigating in the world with their child. *(Social/behavioral)*
Engaging the pediatric patient	With kids, trying to get down on their level, trying to make myself smaller, like sitting down or kneeling on the floor or something like that, try and help them know that I see them. *(Social/behavioral)*
Emphasizing voluntariness	I always emphasize that it’s voluntary and that it won't affect their care at [hospital], just because I know certain families may think that, ‘Well I’m getting care here at the hospital, so I have to join research, or else maybe my care might change.’ I let them know to pause me or stop me at any time. I also stop for any questions they may have, or any concerns, and it doesn't even have to be questions, and to make sure to answer all those questions, while at the same time acknowledging that if they’re a bit iffy on it, I’ll give them all the time they need to think …. I do not want to pressure or make them feel pressured to join the study. *(Clinical research core)*
Social and power dynamics	There certainly is a power dynamic, and it’s one that you have to tread carefully …. I try to kind of consider it as a conversation between two experts. ‘I have an expertise in what the study is about. You have an expertise in your experience and your family, and so you decide what’s right for you, and really at the end of the day, we’re trying to leverage your expertise in order to better be able to serve patients and families in some way.’ *(Social/behavioral)*
Language choice	We’ve been really drilling into how we share information about the study and how we communicate with families, in terms of making sure all of our materials are approachable, are an appropriate reading level, are written such that they can be meaningful to families, regardless of their level of comfort or experience either with medical jargon, or with research, just to ensure that we’re not causing families any discomfort or alienating them just in terms of how we present the research. *(Specialty clinic)*
Describing the study	I feel like it comes down to maintaining communication with the family, making sure that it’s a back-and-forth, pausing for questions that the families might have, verifying that they understand, by having them repeat the information back to you, and then if they don't have any questions, asking them things like what hesitations they might have, or what uncertainties about what I’ve presented to them, and always providing them the option of an out. *(Clinical research core)*
General hesitation about research	There’s definitely been situations where the families are more wary of research and doctors in general, I would say, where perhaps they’ve had a bad experience in the past, so they are more reluctant to trust going forward, and for those kinds of situations, I just try to give the family as much information as possible in terms of the study, answer all their questions as best as I can and if they don't feel the response is adequate, always remind them of how they can connect with the PI [principal investigator] to get those questions answered, and I just try to give them as much space as possible so that they don't feel pressure or obligated to join research, especially if they’re already a bit distrustful of research in general, just because trust is easy to break. *(Clinical research core)*
Motivations	One of the key components in my experience for building rapport and engaging families is explaining both the benefit to them and the benefit to others from the research, like what’s actually gonna happen, right? They’re not just filling out surveys for the sake of doing surveys. *(Specialty clinic)*
Study risks	I definitely don't want to downplay their concerns about risks or side effects, unless it seems like they’re really misunderstanding what those are, but just more focusing on the fact that it’s their choice, if they would like to participate, and that if they do choose to, that they’re not locked into that forever. *(Intensive care)*
Study burdens	People are concerned that it’s gonna take too much time and take away from caring for their child, or doing work. They have a job and doing all these other things …. Sometimes people automatically will be like ‘This is not… I don't…’ ‘cause they don't know what it is yet. ‘I don't think we can do this. We just have too much on the plate.’ *(Social/behavioral)*
**Follow-up**	
Following through	I was recruiting for … a multi-site study where we’re a sub-site, so we’re not the folks who have necessarily all the answers all the time, and so my approach to that is essentially letting them know, ‘You know that is a great question. Let me go ahead and follow up with whoever is the applicable person,’ and then at that point follow up with them and let them know. *(Clinical research core)*
Longitudinal relationship building	We are with these patients so closely and for a long time that they confide things into us and then we are kind of able to escalate concerns… we grow with them, see how things could be better, and then you know speak for them, but we also always encourage them to speak up with anything that they’re not happy about. *(Specialty clinic)*

* Interview code numbers omitted for interviewee privacy.

#### Pre-approach

Some interviewees working in clinical settings spoke about the importance of checking with the primary clinical team before approaching a family to gauge their capacity for research. Reasons for not approaching a family included not meeting inclusion criteria, indications in the medical record of a complex clinical situation, which could indicate a stressful time for the family, or a lack of study materials in the family’s preferred language.

#### Initial Connection

Interviewees described several approaches to connecting with families upon meeting. Most discussed their style or persona; about half of these said they were careful to meet families warmly, calmly, and smiling to convey friendliness, though one noted the importance of matching the family’s energy in light of the clinical context. Several discussed their efforts to find common ground with a family, such as by asking questions to find shared interests. About a third of interviewees described clarifying their role when first approaching a family, for example by explaining the distinction between the research and clinical teams, or sharing information about their department or principal investigator. Several interviewees also spoke about the importance of confirming correct names, pronouns, and pronunciations at this stage. About a third discussed awareness of families’ perception of them based on factors like their attire or age. Several also discussed awareness of how personal biases could affect recruitment interactions and a desire to learn how to better manage these biases; additionally, a few who identified as white noted how the history of white researchers exploiting marginalized groups could influence families having reservations about talking to them. Finally, half of interviewees discussed meeting with families who use languages other than English and highlighted challenges such as additional time needed for recruitment and variable access to translated materials and/or interpreters.

#### Building Connection

These interviews included rich discussions of building relationships. Factors within the relationship-building process included the following: responding to the needs of the family and pediatric patient, considerations to support voluntary decision-making, and communication about study specifics.

All interviewees identified recognizing and accommodating the needs of participants and families as important and necessary for building relationships, reducing barriers, promoting equity, and honoring a family’s autonomy and humanity. Examples included flexibility in study visit scheduling, extra accommodations (e.g., providing refreshments for inpatient families, traveling to collect questionnaires, or finding childcare options), and particular awareness of timing in emergent or intensive care settings, which can be stressful and traumatic. About a third of interviewees discussed the importance of warmly engaging and interacting directly with the pediatric patient. Friendly and direct interactions with the patient, adjusted according to their developmental and cognitive needs, were strategies to empower pediatric patients and recognize their developing autonomy.

All interviewees stressed the importance of explaining the voluntary nature of research to ensure families understand their rights and make an informed decision. About a third discussed voluntariness as a key facilitator in establishing trust by reducing pressure on families and helping address the power dynamic between researchers and families. Interviewees highlighted the importance of recognizing hesitation to participate through non-verbal cues or implicit dissents (e.g., declining participation by continually asking recruiters to return later). When discussing the role of researcher-family power dynamics in relationship building, several emphasized how these dynamics are often compounded for families from marginalized backgrounds. They discussed various ways of addressing power dynamics, including giving families sufficient time to decide, clarifying the distinction between research and clinical care, and emphasizing families’ roles as the experts in their experiences.

Most highlighted the importance of using clear language during the informed consent process for building trust and helping families feel comfortable asking questions and discussing concerns. However, several noted that study materials often include complex medical jargon and vague framings that can alienate families. About half discussed providing as much detail and transparency as possible when describing the study, both to allow families to make informed decisions and to build trust. Several discussed spending extra time with families who were hesitant about participating, sometimes connecting families with investigators or clinicians to learn more. Most interviewees also highlighted the significance of questions throughout the consent process; several noted that being asked clarifying questions indicated a level of trust and that the family was thinking critically about their involvement, while a lack of questions could indicate disinterest or, conversely, familiarity with and general trust in research. Regarding families’ motivations and concerns, several interviewees described forthrightness about expected benefits and potential risks as important for building a trusting relationship. Most also discussed participant burdens and barriers, including transportation, inadequate compensation compared to lost wages, lack of support for childcare, and the disproportionate impact of these barriers on some families.

#### Follow-up

About half of interviewees spoke about following through with participants, for example addressing previously expressed concerns, providing reminders, checking in on clinical needs, and making themselves available for questions. Additionally, just over half discussed the value of building relationships longitudinally, with several noting that studies with only one point of contact can be difficult for building relationships.

### Structural Backdrops

Interviewees identified structural factors that influenced building relationships with families, including factors related to access and diversity, clinical relationships, and the COVID-19 pandemic. Table 4 shows exemplar quotes.

**Table 4.** Structural factors affecting relationship building: codes and exemplar quotes

Factor	Exemplar quote and primary research setting*
Access barriers to research and clinical care	I think there’s a lot of inequity in research. Folks who don't have access to medical care, folks from minority groups, folks with lower socioeconomic statuses, less education, their needs are much less represented. I think of our studies … and a lot of kids whose parents are taking them into [clinic] and they’re getting support, therapy, psychological or physical therapy, and for each of those kids, I’m sure there are many kids out there whose parents aren't even taking them to the doctor, who don't even have the resources to seek out that care. *(Specialty clinic)*
Translation and interpretation	When I’m there [with Spanish-speaking families], there’s no interpreter. It’s just me talking, and I think that makes them feel a lot better .… I introduce myself in Spanish: ‘My name is [Name],’ and then you see their face relax, like ‘Oh, okay. This is gonna be a normal interaction. This isn't gonna be an interpreter interaction and I don't have to pay as much attention. I can let this flow and make it more natural.’ *([Setting omitted for interviewee privacy])*
Incentives and reimbursements	Sometimes I feel like it’s just the nature of the research study itself. It’s complex. It asks a lot of participants. We give them very little compensation in return for their time. … I feel like we mostly end up recruiting people who are pretty motivated from the moment they come in. *(Social/behavioral)*
Prioritizing participant diversity	Anyone who’s been sort of marginalized or underrepresented in research and basically non-white, lower SES [socioeconomic status], people who don't tend to have as loud of a voice as white people, and it may be because they are not being asked or reached out to. *(Social/behavioral)*
Diversity of research teams	Sometimes I have to take off my research coordinator hat and I just have to put on a human hat to just connect to the parents where they are. … This is where a team is extremely helpful, ‘cause I worked before where I was the only person in the team and that was extremely difficult to put on different hats and just give myself a different perspective. So I think just having a well-represented team makes a difference. *(Social/behavioral)*
Community stakeholder involvement	We actually have kids, like where we built rapport with those in our community to look at our drafts of the documents. We did a lot of that and that was really helpful to really hone in on whether this document would be efficiently used when sharing it with research participants. *(Specialty clinic)*
Research oversight pragmatics	I really get to the bones of the study very quickly because, unfortunately, our consents are 15 pages long, and it’s a challenge. When somebody’s got a sick kid, they’re not necessarily gonna read the whole consent and I know the IRB [Institutional Review Board] doesn't want to hear this, but you’ve got to make sure that they get the information. *(Intensive care)*
Prior relationship with clinician or institution	A lot of these kids have illnesses where they’ve been in the [unit] numerous times, so they’re used to it. Obviously not all of them are like that, but [hospital] obviously has a reputation that they’re one of the best children’s hospitals in the country, so they automatically have the trust. … I don't think it matters whether I’m a doctor or research coordinator. They’re willing to listen. *(Intensive care)*
Streamlining research in clinical care	Often I’m like sitting by a family in a waiting room, or trying to jump in the room between providers and it just feels like research is an afterthought. Here we’re trying to say how important it is, but I’m like, ‘Wait. Okay, let me rush you through the consent process before the doc—’ They’re here to see the doctor and their time is valuable, so I do not want to take the time away. *(Specialty clinic)*
Clinician/chart facilitation of research	If I’m meeting a patient and family and their medical provider that they’ve worked with for years says, ‘Oh you have to meet [Name]. She’s a great part of our team. She’s doing this interesting research. You can decide whether you think it’s interesting also.’ Those warm handoffs can be so powerful. *(Social/behavioral)*
Interactions with other research studies	If you’re reaching out to a family and don't know they’ve already been approached by two other studies in the last three days, then a family might just be like, ‘Look. We’re so overwhelmed. People have already asked us this. We’re not interested,’ whereas if there’s someone who is more able to manage that and see exactly who is approaching what families, then I think that sort of gives everyone a better opportunity for success, and it helps families. *(Intensive care)*
Impact of COVID-19 pandemic	Coming to a research study, it’s not a mandatory medical appointment, and I thought about how families would be comfortable to come in for an in-person visit, but the trust that they have shown in our teams has been incredible. I haven't had a single person that I’ve contacted has said ‘No. This is COVID times. We don't want to visit.’ Everybody has gotten back to me saying, ‘We have done this research with you. We know that you will take all the necessary precautions.’ I email them saying ‘We’re full PPE [personal protective equipment]. This is what we’re going to do. We have Plexiglass.’ *(Social/behavioral)*

* Interview code numbers omitted for interviewee privacy.

Many interviewees discussed factors affecting access to research, including translation into languages other than English and Spanish, inadequate incentives or reimbursements, childcare needs, transportation needs, and ability to obtain referrals to research through specialized care. About half noted a distinct lack of diversity among participants, particularly across dimensions of race, ethnicity, and language, and several discussed the need to diversify research teams to better reflect the patient population. Some interviewees spoke about community stakeholders being involved in study design or providing feedback on their participation, and one noted that a lack of community involvement made them feel the study’s goals were disconnected from the community it aimed to serve. About half discussed research oversight, sometimes as creating challenges (e.g., for translating consent materials or providing incentives), but also as assuring families that studies were appropriately monitored.

More than half of interviewees discussed how a family’s prior clinical relationships or past experiences with the institution, including its reputation, could facilitate research relationship building and sometimes motivate – or discourage – participation. Most also discussed the role of clinicians and/or medical chart access to support recruitment, although some recalled being asked not to approach a family for medical or social reasons even if the patient was otherwise eligible. Half of interviewees described additional challenges with the integration of research and clinical care, describing a lack of scheduled research visits in some clinical settings and a need to defer to clinical activities. One noted the importance of increasing research presence and strengthening research-clinical ties in satellite clinics. Several interviewees commented that families sometimes felt stressed by multiple requests for different research studies.

Finally, more than half of interviewees identified new challenges during the COVID-19 pandemic. These included difficulty seeing non-verbal cues while recruiting remotely or while donning personal protective equipment (PPE), disruptions to families’ experiences due to limitations on clinical visitors, increased difficulty accessing interpreters, need for greater scheduling flexibility to accommodate families’ increased logistical barriers, adjusting to and standardizing virtual consent processes, and increased hesitancy about participation due to families’ concerns about in-person visits, for example for children unable to wear a mask for the duration of a study visit. Approaches to relationship building during the pandemic included increasing consistency with email communications, gauging interest by speaking with healthcare providers, using video conferencing to allow for unmasked face-to-face interactions, printing a photo of oneself to share when wearing PPE, and giving families space to discuss their challenges.

## Discussion

Interviews with research staff revealed that factors at the individual, relational, and structural levels influence relationship building with potential participants and their families. Across these levels, four overarching themes became apparent: (1) staff experience tensions as well as supportive relationships with various actors, which influence their ability to perform tasks and address challenges; (2) integration with clinical teams can facilitate relationship building, but it is also important to clearly distinguish research from clinical activities; (3) voluntariness is seen as foundational to obtaining valid consent as well as building trusting relationships; and (4) contributors to inequities and access barriers are prevalent at multiple stages of research.

First, staff saw themselves as variably supported by or in tension with other individuals and groups, including other coordinators, investigators, and institutional priorities and processes. Prior work has shown that research staff see themselves as advocates for multiple perspectives – clinical patients, research participants, and the research study – simultaneously [15]. Consequently, staff sometimes must navigate conflict between patient care and study requirements [16]. Our interviews support these findings and illustrate numerous actors and dynamics that facilitate or impede their advocacy roles. For example, staff noted that they sometimes had different priorities from their principal investigators or had to navigate complex institutional processes. As staff are well positioned to observe the impact of such priorities and processes on potential participants and their families, it is important that they be empowered to describe barriers that families experience to their teams and have a voice in institutional policy development.

Second, staff described the importance of being connected to clinical teams while also distinguishing research from clinical care. Staff highlighted the value of a “warm handoff” (i.e., a known clinician introducing a family to the research team) for forming an initial connection, as well as how positive prior relationships with the clinical team and/or institution can facilitate research – and, conversely, how a limited relationship, lack of clinical integration, or negative past experiences can limit or encumber research engagement. These findings build on prior work showing that many parents prefer to learn about research from their clinical care team [2] and that clinical experiences can influence patients’ views about research participation in an integrated setting [17]. While discussing the importance of, and the need to streamline, clinical research integration, our interviewees were also careful to clarify the distinction between the two, illustrating the key role of research staff in maintaining ethical boundaries between research and clinical care [18]. Embracing this role could present opportunities for staff to identify and address other ethical concerns at the clinical research boundary, such as the therapeutic misconception (i.e., not understanding that the goal of research is to produce generalizable knowledge rather than to benefit research participants) [19]. This topic was not explored in these interviews but could be a focus of future work.

Third, staff strongly emphasized the importance of voluntariness and the multiple measures they took to ensure voluntary choices. Voluntariness was described as key to trust building. By alleviating pressure for families, staff gave families space both to make a decision that was right for them and to feel that they could trust the research team. As several of our interviewees noted, and has been discussed in the setting of the neonatal intensive care unit [20], this may be particularly important in stressful clinical contexts.

Fourth, our interviewees discussed multiple examples of inequities and access barriers that introduce potential for disparities in research. For example, many staff noted challenges related to translating research materials into other languages. Research staff are uniquely situated to observe a wide range of barriers that may be revealed during the relationship-building process. While their perspectives alone cannot offer a complete picture, they represent an invaluable bridge between the experiences of participants and the research institution, and a deeper understanding of their observations is warranted.

### Limitations

This study described perspectives of staff working in one pediatric research institution and its affiliated academic medical center; staff working in other institutions may identify additional considerations and priorities. While we spoke with staff working across a range of pediatric clinical research settings, there may be additional viewpoints not represented in our sample, for example, from those working with adolescent patients in different clinical contexts, especially when the patient and their family may have different perspectives about participating. Furthermore, the staff we interviewed were primarily female, non-Hispanic white, and highly educated, and thus reflect limited perspectives on recruitment. Nevertheless, the depth of reflection and engagement by these interviewees provides a robust description of their perspectives. Future work should build on our findings and evaluate needs for research staff across a broader representation of the profession. This study was limited to research staff because of the absence of their voice in the extant literature; other stakeholders may, and likely do, have different perspectives and priorities. Future work must assess how these themes correlate with views of other stakeholders, most importantly parents or legally authorized representatives tasked with making the enrollment decision and pediatric patients themselves. This study should also be contextualized by its timing during the COVID-19 pandemic. While we were able to conduct robust remote interviews using videoconferencing tools that were familiar and easily accessible to our study population, we acknowledge the potential impact of the broader context on our recruitment, data collection, and interpretation. Finally, the authors acknowledge that our role as researchers, some working in the same institution as the staff who were interviewed, informs our interpretation of the data. To ensure the trustworthiness of our analyses, we incorporated the member checking process to validate our preliminary findings, and we involved authors from a variety of institutions and types of roles throughout study development, data collection, and interpretation.

## Conclusion

Pediatric research staff play a critical role in supporting families of potential participants and recognizing ethically important issues during recruitment such as access barriers, threats to autonomy, and other needs of patients and their families. Efforts to improve research recruitment can build on the framework of staff perspectives on research relationship building presented here, and future work should aim to empower staff to fulfill their role as advocates for prospective participants and their families.

## Supplementary material

For supplementary material accompanying this paper visit https://doi.org/10.1017/cts.2022.469. click here to view supplementary material
