# Supplementary material for: Relationship building in pediatric research recruitment: Insights from qualitative interviews with research staff
Source: J Clin Transl Sci. 2022 Oct 3;6(1):e138. doi: 10.1017/cts.2022.469 (PMC9794958; doi:10.1017/cts.2022.469)
Supplement: Supplementary file 1 [file ctssup.zip › S2059866122004691sup001.docx]

**Supplementary Material. Codes and definitions**

| **CODE** | **Definition/example** |
| --- | --- |
| **Individual** | These codes all focus on comments specific to the individual CRC, as opposed to comments focused on the relationship between the CRC and participant/family or structural elements related to the recruitment process |
| 1. Developing recruitment skills | How CRC developed recruitment skills and/or learned how to interact with patients/families |
| - 1. Training | Formal training to help CRC develop recruitment skills and learn how to interact with patients/families; also includes training CRC would like to have, e.g., communication, EDI/cultural sensitivity, trust building |
| - 1. Experience | CRC’s professional experiences, including shadowing, that helped them develop recruitment skills and/or learn how to interact with patients/families, whether in a CRC role or otherwise [discussion of personal experiences and motivations to be coded as “Personal motivation/personal experiences”] |
| 1. Perception of role | How CRC perceives their professional role, for example within the study team or with respect to engagement with potential/current participants; includes discussion of role in relation to, and power dynamics with, PI |
| 1. Personal motivation/personal experiences | CRC’s own personal life experiences that may influence their approach to their job or their role on the study team; CRC discusses their personal motivation or values in the context of their role as a CRC. Examples could include a commitment to advancing science, promoting equity, supporting career advancement among CRCs, or working with particular populations |
| 1. Other | Other “Individual” that doesn’t fit in codes 1 through 3 |
| **Relational** | These codes all focus on comments specific to the relationship between the CRC and participant/family, as opposed to comments focused on the individual CRC or structural elements related to the recruitment process |
| **Pre-Approach** | Relational issues that arise for CRC prior to approaching a potential participant/family about a research study |
| 1. Judging when/whether to approach | Deciding when, how, and/or whether to approach a patient/family about research participation |
| 1. Other | Other “Pre-Approach” that doesn’t fit into code 5 |
| **Initial Connection** | Relational issues that arise during the initial meeting/interaction with a potential participant/family, generally before the consent conversation |
| 1. Approach to communication | Discussion of approaches to communication, including pragmatics/logistics and attention to family’s needs in approach, e.g., phone/text, strategy when entering a room, deciding whether to introduce research at first meeting |
| 1. Clarification of role | Clarification of the research staff member’s role in relation to the potential participant, including distinction between research team and medical team, research staff and PI, etc. |
| 1. Names, pronouns, pronunciation, etc. | Discussion of names, pronouns, pronunciation in the context of building rapport with participant/family (act of introducing themselves would be coded as “approach to communication”) |
| 1. Style/persona | Use/visibility of style/persona in the context of building rapport with participant/family (e.g. formality, authenticity, reading the room, making conversation, etc.) |
| 1. Finding common ground | Attempt to find common ground with participant/family in the context of building rapport |
| 1. Families who use language other than English | Considerations for potential participants/families who use a language other than English in the process of making an initial connection; includes discussion of language concordance |
| 1. Family’s perception of coordinator | Discussion of how families perceive the coordinator and how that impacts the ability to build relationships and trust (e.g. racial/ethnic concordance; assumptions related to age; making judgments about the coordinator) |
| 1. Personal biases | Attention to personal biases held by the coordinator in initial connection with participants/families |
| 1. Other | Other “Initial Connection” that doesn’t fit into codes 7 through 12 |
| **Building Connection/Give and Take** | Relational issues that arise after the initial interaction related to building a connection with a potential participant/family in the context of the potential research study, generally during or after the consent conversation |
| 1. Attention to family context/needs | Paying attention to (or being respectful of) the family’s time and other contextual features, such as their clinical needs or family obligations throughout the ongoing interaction, or remembering personal details about the family |
| 1. Power/social dynamics | Power or social dynamics, including belief that families hold the power or acknowledgment of complexity of power dynamics, and how they play into relationship building with participants/families |
| 1. Voluntariness | Emphasis/focus on the voluntariness of research, including issues around enrollment decision not affecting clinical care and responding to “soft refusals;” paying attention to non-verbal cues |
| 1. Language choice | Wording chosen by participants/families for recruitment discussion/materials, for example whether materials are accessible to non-scientists |
| 1. Motivations | Reasons for being interested in research, generally or specific study; may include discussion of research benefits |
| 1. Description of study | Explanation of study procedures/informed consent information/what the participant needs to do, including potential participant asking clarifying questions or not |
| 1. Study risks | Considerations about study risks |
| 1. Burdens of participation | Considerations related to the burdens of participation on the part of the participant, including ways of overcoming barriers. Includes factors such as time required to participate; extra hospital or clinic visits; extra tests; increased calls from staff |
| 1. General comfort/discomfort with research | Barriers or facilitators related to general comfort level with research, including research privacy concerns and including references to “guinea pigs” |
| 1. Assent/engaging with the pediatric patient | Assent/engaging with the pediatric patient |
| 1. Other | Other “Building Connection/Give and Take” that doesn’t fit into codes 14 through 23 |
| **Following Through** | Relational issues that arise after initial connections and discussions about the study; may include issues both before and after decision to participate |
| 1. Following through | Discusses following through, keeping promises, doing what you say you’ll do when you said you would, etc. |
| 1. Longitudinal relationship building | Longitudinal relationship building, includes discussion of building trust over time or participants forgetting prior interactions; ongoing communication via newsletter to participants; how to respond after a prior negative interaction |
| 1. Other | Other “Following Through” that doesn’t fit into codes 25 or 26 |
| **Structural** | These codes all focus on comments specific to the structural elements related to the recruitment process, as opposed to comments focused on the individual CRC or the relationship between the CRC and participant/family |
| 1. Translation, interpretation, and language | External factors related to translation, interpretation, and language that affect the ability to include patients/families who use a language other than English |
| 1. Incentives/reimbursements | Barriers or facilitators related to incentives/reimbursements for research |
| 1. COVID-specific barriers | Barriers to relationship-building stemming from the COVID-19 pandemic, including reflections on changes due to COVID-19 even if not mentioned explicitly (e.g., shift from in-person to phone) |
| 1. Access to research participation/care | Barriers to building relationships related to access (or lack thereof) to clinical care or research opportunities |
| 1. Research oversight pragmatics | Issues related to research oversight. Includes challenges with IRB speed/process; support by IRB and others. |
| 1. Participant’s prior relationship with clinician/institution | Participant’s prior relationship with clinician, clinical/specialty team, and/or institution, including discussion of institutional reputation and including prior clinical relationships with research staff (NOTE: prior research relationships should be coded as Longitudinal relationship building) |
| 1. Clinician/chart facilitation of research | Need for clinicians to facilitate research and research relationships, including awareness of clinical information through the patient’s chart. Includes discussion of the idea of clinicians as gatekeepers to patients in the research recruitment and consent process |
| 1. Streamlining research in clinical setting | How research fits into the clinical setting, e.g., timing research appointments with clinical appointments |
| 1. Interactions with other research studies | How CRC’s studies interact with other research studies. Includes participants being recruited for, or being eligible for, multiple studies |
| 1. Community involvement in research | Involvement of the community in research, including general community engagement, involvement of community members in study design/implementation, etc. Includes discussion of community involvement strategies (effective or ineffective) and lack of representation of certain perspectives/voices in research generally or a specific study. [Discussions about participant diversity within studies should be coded as “Prioritizing participant diversity”] |
| 1. Prioritizing participant diversity | Need/desire to prioritize participant diversity in research, including attention to participant race, ethnicity, gender, SES, rural settings, disability or cognitive impairment, etc. Includes discussion of context of historical research abuses. |
| 1. Diversity of research team | Diversity (or lack thereof) within the CRC cohort and/or PIs |
| 1. Other | Other “Structural” that doesn’t fit into codes 28 through 40 |
| **Response to “how do you show respect”** | Use this code to capture CRC responses to the question “how do you show respect” |
| **Response to “how do you build trust”** | Use this code to capture CRC responses to the question “how do you build trust” and follow-ups about signs of trust/lack of trust, examples of successful trust-building, etc. Code each sub-question separately. |
| **Interviewee characteristics** | Includes: role (types of studies; study population; daily activities); time at current job; total time as research staff; age; gender; race/ethnicity; highest degree. Most likely to be used at beginning and end of interview. Any discussion of training should be coded as Individual>training. |
