# Supplementary material for: Relationship building in pediatric research recruitment: Insights from qualitative interviews with research staff
Source: J Clin Transl Sci. 2022 Oct 3;6(1):e138. doi: 10.1017/cts.2022.469 (PMC9794958; doi:10.1017/cts.2022.469)
Supplement: Supplementary file 1 [file ctssup.zip › S2059866122004691sup002.docx]

**Appendix. Interview guide**

*[Introductory and consent language omitted]*

*[NOTE: Sub-bullets are optional prompts for follow-up as needed]*

**Warm-up**

- Can you tell me a little bit about your current role on your research team?
  - What types of activities do you do on a day-to-day basis?
  - What kinds of patients or families do you most often work with?
  - What types of studies are you most often involved with?
  - What are some things you like about your job?
  - What are some things you find challenging about your job?
- Can you tell me about how you learned how to interact with patients and families in a research context?
  - What have your current or former PIs done to foster your learning?

**Effective communication**

- Now I’d like to talk about how you communicate with families during recruitment. What is your approach to communication when you meet a new family?
- I would also like to consider potential participants who you don’t end up approaching. Can you tell me about a situation when you decided not to approach a potential participant who was otherwise eligible?
  - What sorts of things might dissuade you from approaching someone even if they are eligible for the study?
- Many researchers are trying to improve equity, diversity, and inclusion within their studies. Have the studies you have been involved with tried to incorporate these goals into their protocols? Can you tell me how they have tried to do that?
  - What efforts have been most successful?
  - What efforts have been less successful?
  - In what ways have you incorporated these goals into your communication with families?
  - In your experience, how do an organization’s values influence your interactions with families?

**Respectful relationships**

- Now I’d like to know more about your experiences building relationships with research participants and their families. How do you demonstrate respect when recruiting prospective participants and families?
  - How do you think about the power dynamic between you as a member of the research team and the person you are recruiting?
- How do you seek to build trust with prospective participants and families during the recruitment process?
  - How easy or difficult is it for you to build trust during these conversations?
  - What are some signs that a participant or family trusts the research study? What are some signs that a participant or family does not trust it?
  - Tell me about a time when you were able to form a trusting relationship with a participant or family.
    - What factors allowed you to do that?
  - Tell me about a time when you were not as successful forming a trusting relationship.
    - What factors made that difficult?
- What is the biggest barrier you face in building trust?
  - What makes this difficult?
  - What has helped you overcome this barrier? What other things might help you overcome this barrier?
  - What other barriers do you face? What would help you overcome these barriers?
- Tell me about a time when you recruited a family from a different cultural background than yours.
  - What, if anything, was different about your approach?
  - What challenges did you face in that situation?
  - What would help you feel more prepared for a similar situation in the future?

**Committed partnerships**

- My next few questions are about how research studies incorporate patient and family priorities and needs. This might include anything from the types of questions that are asked, to how the studies are set up, to whether and how findings are shared. To what extent do you feel like the research studies you recruit for address the priorities and needs of patients and their families
  - What strategies have you seen be effective to bring in patient and family voices?
  - Are there specific groups or communities whose needs are not well represented?
    - What impact does this lack of representation have on your work?
- To what extent are community members part of advising on, planning, or implementing the studies you are involved with?
  - In what ways have you seen this approach be effective in helping the study to better address the priorities and needs of patients and their families?
  - In what ways could community involvement be improved?

**Methods to resolve problems**

- Now I have a few questions about how you respond when families raise questions or concerns during recruitment. How would you approach a situation where a family is questioning whether to participate?
  - How do you typically respond when a family raises concerns about the risks or side effects of the research?
  - How do you typically respond when a family seems uncomfortable with the idea of research or indicates they don’t trust research?
  - What might indicate that a family didn’t understand something about the study?
    - How do you typically respond in these situations?
  - What do you do when a family seems interested but is facing access barriers that may prevent them from participating?
    - What tools or approaches are most valuable in these situations?
- What additional barriers to building trust, if any, have come up since the start of the COVID-19 pandemic?
  - How have you navigated these?
  - To what extent have you noticed if different populations tend to face different barriers?

**Sustainability**

- Now I’d like to ask you to think about what systematic changes would help improve the recruitment process. What tools or resources (e.g., processes, policies, resources) would be helpful for you to improve your interactions with families during the recruitment process?
  - How would these help overcome the barriers you mentioned?
  - What impact would these tools have on your work in terms of increasing the involvement of underrepresented groups in research?
- How can research institutions best support research staff in building trusting relationships with families?
  - What systems or structures would you change to improve your ability to recruit people from diverse populations?

**Interviewee demographics**

Thank you so much for taking the time to talk with me today. I have just a few last demographic questions before we wrap up.

- How long have you been in your current job?
  - Have you had other research staff jobs before this one, including at other organizations? If yes, how long have you been working as a research staff member, including your current job?
- What is your age?
- What is your gender?
- How would you describe your race or ethnicity?
- What is the highest degree you have earned?
- What trainings or certifications have you completed that are relevant to your current role?
  - Have you taken any trainings specific to equity, diversity, and inclusion?

**Wrap-up**

Before we wrap up, is there is anything else I should have asked you about that I didn’t ask about?

That’s the last question I have for you. Do you have any additional thoughts or questions for me?

*[Conclusory language omitted]*
